# Physiotherapists’ Evidence-Based Practice profiles by HS-EBP questionnaire in Spain: A cross-sectional normative study

**DOI:** 10.1371/journal.pone.0269460

**Published:** 2022-06-03

**Authors:** Juan C. Fernández-Domínguez, Joan E. De Pedro-Gómez, Rafael Jiménez-López, Natalia Romero-Franco, Ana B. Bays Moneo, Ángel Oliva-Pascual-Vaca, Albert Sesé-Abad

**Affiliations:** 1 Care, Chronicity and Health Evidence (CurES) Research Group, Balearic Islands Health Research Institute (IdISBa), Palma, Spain; 2 Department of Nursing and Physiotherapy, University of the Balearic Islands, Palma, Spain; 3 Balearic Islands Health Research Institute (IdISBa), Palma, Spain; 4 Department of Psychology, University of the Balearic Islands, Palma, Spain; 5 Department of Health Sciences, Public University of Navarra, Navarra, Spain; 6 Department of Physiotherapy, Faculty of Nursing, Physiotherapy and Podiatry, Universidad de Sevilla, Sevilla, Spain; 7 Escuela de Osteopatía de Madrid, Madrid, Spain; The Education University of Hong Kong, HONG KONG

## Abstract

Evidence-Based Practice (EBP) is a cost-effective approach for improving the quality of clinical care and implementing only well-tested evidence. Health professions, especially physiotherapy, must embrace EBP principles. This paper presents normative data from the Spanish physiotherapist population using the Health-Sciences Evidence-Based Practice questionnaire and explores EBP clusters/profiles of professionals in practice. An intentional sample of 419 practicing physiotherapists was recruited from the Spanish Professional Council of Physiotherapy. Participants completed a cross-sectional online survey with 60 Likert items (scale 1–10) measuring 5 dimensions: 1) Beliefs and attitudes, 2) Results from literature, 3) Professional practice, 4) Assessment of results, and 5) Barriers and Facilitators. The protocol also included sociodemographic, training, and practice-related contrast variables. Normative data were estimated and tabulated for each dimension and then a K-means clustering procedure was implemented using the contrast variables. Results for normative data showed, in descending order, the following 50^th^ percentile values for the five EBP factors: Beliefs and attitudes (8.25), Professional practice (8.00), Assessment of results (7.42), Results from literature (6.71), and EBP Barriers and Facilitators (5.17); all expressed on a scale of 1 to 10. Academic degree, EBP training level, and work time shared in healthcare activity, research, or teaching activity were all statistically significant for discriminating EBP dimension scores. Finally, six different clusters showed that when EBP level is low, the scores in all dimensions are equally low, and vice-versa. The EBP dimensions "Beliefs and attitudes", "Professional practice", and "Evaluation of results" obtained better normative scores overall than "Search for bibliographic evidence and its inclusion in practice" and especially "Perception of EBP barriers", which had the worst score. Normative data are useful for comparing individual scores and the reference population, and information about clusters will enable appropriate global EBP intervention programs to be designed and implemented.

## Background

Evidence-Based Practice (EBP) is an innovative approach for providing health care that seeks to improve the quality and safety of clinical care and to reduce its huge costs in developed countries [1]. Thus, health professions in general have been urged to embrace the principles of EBP, particularly Physiotherapy [2, 3]. In this context, EBP assessment is generally carried out using self-reported instruments, and scientific literature shows a great number of tools have been used for over 15 years [4–7].

Some systematic reviews on the subject have been published, such as the most recent one that analyzed 24 instruments focused specifically on the evaluation of EBP in Physiotherapy [8]. However, all the instruments reviewed revealed significant shortcomings regarding appropriate EBP construct operationalization. The main drawbacks are basically found in the partial nature of the measures and in the non-inclusion of aspects related to the EBP process itself, such as assessment of results and implementation of evidence. Further, it is important to note that many of the proposed instruments do not present sufficient quality evidence of the psychometric behavior of their items, their latent structure, or the rest of the sources of validity evidence required by the international standards in use [9, 10]. Finally, only some of these instruments were built with a transdisciplinary approach, although the usefulness of and interest in having a standardized EBP measure that enables comparisons between different health sciences professions had been highly recommended [11].

The Health-Sciences Evidence-Based Practice Questionnaire (HS-EBP) was developed and validated to cover these gaps in EBP construct measurement [12]. This tool includes a comprehensive theoretical analysis and operationalization of the EBP construct and provides enough quality evidence of psychometric validity in accordance with the requirements of the international standards for test development and validation [9, 10]. The HS-EBP questionnaire operationalizes a definition of the complete EBP process from the search for evidence to the evaluation of the results of its implementation in practice; it includes the measurement of barriers/facilitators; and is also designed to be administered to all Health professions. All these characteristics mean that the HS-EBP can be used to evaluate the impact of specific interventions to improve EBP in Health professionals [12] and even to serve as a guide for EBP management within the curricula of health-related university studies. More specifically, this tool can be useful for assessing physiotherapists because it meets the requirements of the World Physiotherapy accreditation system.

As the HS-EBP questionnaire developers, an important step in its validation process is to provide normative data about the different target populations it is intended to measure. Normative data are appropriate and even necessary for a correct interpretation of the test scores obtained from questionnaires developed within the framework of Classical Test Theory (CTT) [9]. A CTT score does not have an absolute meaning but rather is relative to its reference population normative data; that is, a person’s test score can only be correctly assessed if normative data are available from the reference population to which that person belongs. Standards 7.2, 7.4, and 10.8, included in the current "Standards for Educational and Psychological Testing", highlight the relevance of obtaining normative data for test score interpretation.

However, although providing normative data is relatively common for measuring health-related constructs [13, 14], to our knowledge, there are no normative data on EBP for the profession of Physical Therapist [8]. EBP normative data for the physiotherapist population can provide information for comparing different practice settings, healthcare organizations, and/or health services, making it possible to improve both student training programs and professional practice and, as such, contribute to optimizing management of the Health System [5]. If we also take into account the fact that it is difficult to establish criterion-referenced measures on EBP test scores a priori, providing normative data may be a good starting point for obtaining a context-fitted interpretation of physiotherapists’ EBP scores. Normative data enable us to explore the HS-EBP dimension scores and find groups of physiotherapists with different EBP profiles. In turn, these clusters can be described by other EBP related variables.

Hence, this study is aimed at providing normative data on the HS-EBP questionnaire with Spanish physiotherapists as the reference population. A second goal is to estimate the differential effects of sociodemographic variables, professional training, and practice settings on the HS-EBP dimension scores. Finally, the existence of different EBP patterns is explored using cluster analysis.

## Methods

### Design and procedure

A cross-sectional online survey design implemented with the “LimeSurvey” platform was conducted between March and December 2019. The survey’s usability and technical functionality were carefully developed and checked by an external software developer company, whilst considering ergonomic factors for its layout (disabilities, devices, etc.). The survey consisted of 11 pages (screens); the first screen included information concerning the purpose of the study, informed consent, data handling and data protection (anonymity), approval by the Balearic Islands University Research Ethics Committee, instructions for completing the survey, and the expected time for doing so. Finally, participants were informed about where the responses were to be stored and for how long. Participants were also invited to contact the research team in case of doubts or technical problems during survey completion. The next ten screens gradually included sociodemographic, organizational, and EBP-related variables with four or five items per screen, and the items belonging to each dimension of the HS-EBP questionnaire with 14–15 items per screen.

A survey link was sent by e-mail to the Spanish Physiotherapy Professional Council in order to include the nine regional delegations with the largest number of affiliated physiotherapists. Participation was entirely voluntary and informed consent was assumed when a physiotherapist completed and sent off the answers. At all times (before the final submission), participants were able to review and change their answers through a Back button. Likewise, a cookies system from the LimeSurvey platform was used to prevent multiple entries from the same participant. Participation rate was 51.5%, while the completion rate of the survey was 46.5%.

Uncompleted questionnaires were removed from the analysis and no incentives were offered for participating in the study. All survey data were automatically processed by the platform and stored on an external device with a password-protected server. All information was always in the custody of the research team members.

Methods and results are reported according to the Checklist for Reporting Results of Internet E-Survey [15] and the checklist requirements of the STROBE Statement for cross-sectional studies were met [16].

### Sample

The Spanish Professional Physiotherapy Council is composed of 17 regional delegations corresponding to the 17 Spanish Autonomous Communities, with a total number of 54,258 physical therapists (reference population). The eligible population was made up of delegations with the largest number of inhabitants and physical therapists according to the data reflected in the 2018 Spanish Statistics Institute report [17]: Andalusia (7,129), Aragon (1,533), Catalonia (10,252), Castile and Leon (2,586), the Valencian Community (5,176), Madrid (10,581), the Balearic Islands (1,302), Galicia (2,772), and the Basque Country (2,761); representing a total number of 44,092 physiotherapists, 82% of the reference population. Both the reference and eligible population presented the same proportion for females (64%) and males (36%). Regarding the nine selected delegations, the proportion of males ranged from 28% to 40%, but no statistical differences were detected between any comparison (p = .64); with comparisons between female proportions (complementary proportion values 60%-72%) also turning out to be statistically non-significant (p = .60). The age group distribution of the physiotherapist population showed that 49% were under 35 years old (n = 21,811), 36% between 35 and 44 (n = 16,030), 10% between 45 and 54 (n = 4376), and 2.9% between 55 and 64 (n = 1260), while the remaining 1.4% was 65 years or over (n = 615).

A non-probabilistic intentional sampling of volunteers was used for extracting a representative sample of practicing physiotherapists. Inclusion criteria were: (a) holding a Physical therapy degree, and (b) currently practicing Physical therapy in Spain. Collaboration of the Spanish Professional Physiotherapy Council was requested, since professional practice in a clinical setting requires mandatory registration. An invitation to collaborate was sent to every regional delegation throughout the country. Three gentle reminders were made throughout the study to try to improve the response rate; the first by phone contact to delegations in which no response had been obtained via e-mail. Finally, two other reminders were made via email, the first approximately three weeks after the phone contact, and the second two weeks later. Each regional delegation that agreed to participate in the study undertook to send it via email to all its registered members.

### Instruments

The HS-EBP is a self-reported questionnaire composed of 60 items with a Likert scale response format (from 1 “Totally disagree” to 10 “Totally agree”). Its latent structure contains 5 factors: “Beliefs and attitudes” (F1, 12 items, range: 12–120 points); “Results from literature” (F2, 14 items, range: 14–140 points); “Professional practice” (F3, 10 items, range: 10–100 points); “Assessment of results” (F4, 12 items, range: 12–120 points); and “Barriers and Facilitators” (F5, 12 items, range: 12–120 points) [18]. The instrument has shown reliability and validity evidence from different sources through several psychometric sampling trials. Specifically, content validity evidence obtained an Item Content Validity Index (I-CVI) of ≥80% with regard to the degree of adequacy of the items to their dimension from a sample of 32 EBP experts, including nine physiotherapists [18]. Likewise, in a sample of 869 Health Science professionals from all the Autonomous Communities of Spain, where 97 (19.83%) were physiotherapists, evidence of high internal consistency was obtained, with Cronbach’s alpha coefficients of .93, .96, .84, .94, and .91, for the five dimensions, respectively. Evidence of latent structure validity using confirmatory factor analysis (CFA) was obtained, providing good overall and analytic fit (χ^2^/df = 2.89; RMSEA = .049, with CI90% RMSEA = [.047-.050]; SRMR = .067; and CFI = .99) for the five-factor model, compared to a basal one-factor model and other more complex competing models. Evidence of validity in terms of relationships with other variables obtained adequate results regarding the hypothesis in respect of the following criteria: “Dispositional resistance to change” (negative), “Burnout” (negative), and “Quality of professional life” (positive). Convergent validity evidence to other EBP instruments such as the Evidence-Based Practice Questionnaire (EBPQ) was also confirmed. Finally, the “prior level of training of subjects in EBP” was used as a gold standard for obtaining evidence concerning decision validity [10]. Recently, normative data from a representative sample (n = 443) of the Spanish Osteopathic Professional population were provided using the scores from the HS-EBP questionnaire [19].

The protocol also included the following variables to implement differential analysis: sex, age, academic degree (Diploma/Bachelor’s, master’s, doctorate), number of hours of professional practice, EBP training (yes/no), and EBP training level: Basic (having done an/some introductory course/s to EBP, bibliographic search in electronic databases or similar); Intermediate (in addition to basic, also having done an/some introductory course/s to research methodology: asking a research question, critical reading of scientific articles, interpretation of statistical results, or similar); or Advanced (in addition to intermediate, also having done a/some training course/s on research: statistics and handling computer programs e.g.: SPSS, R, Stata; writing scientific articles, or similar).

Other context variables are practice setting (rural, urban with fewer than 50,000 inhabitants, or urban with more than 50,000 inhabitants); main workplace (“Specialized care”, “Primary/Community care”, “Socio-Health center”, “School system”, “University academic staff”, or “On their own”); type of organization (Public, Private, Mixed); weekly working hours; work time shared in different in daily practice activities (Healthcare activity, Research, Teaching, Management); working alone or as part of a team (yes/no); accredited educational center (yes/no); and whether the professional supervised physiotherapy students in clinical work practice (yes/no).

### Statistical analysis

Preliminarily, several proportion comparison tests for independent samples were used to analyze the degree of the sample’s representativeness in terms of the reference population, for both sex and age groups. A descriptive univariate analysis of the sociodemographic, training, and practice variables was performed using absolute and relative frequencies for qualitative and ordinal variables. Basic descriptive statistics (Mean, SD, 95% Mean CI) were used for quantitative variables and the Kolmogorov-Smirnov test with Lilliefors correction was used to test normality together with the 95% CI of the Skewness and Kurtosis indices (Bliss g_1_ and g_2_).

Cronbach’s alpha (α), Omega coefficient (ω), and Hierarchical Omega (ω_H_) were estimated as reliability indicators of the HS-EBP questionnaire 5-factor latent structure. All these indices are indicative of adequate reliability with values equal to or greater than .80. Percentage of explained variance (% EV) was also computed for each factor. Post hoc power of the proportion comparison test was estimated using G*Power software.

Normative data of HS-EBP factors were estimated by establishing the centiles of the entire distribution. Subsequently, differential analyses of the HS-EBP factors using the EBP-related variables included in the survey protocol were implemented. Categorical variables were analyzed using the Chi-square test and Cramer’s V coefficient (Φ_C_); quantitative variables through independent samples t-test for dichotomous variables; and ANOVA test with Bonferroni multiple comparisons for polytomous ones. The non-parametric Spearman coefficient was used because most of the variables showed non-normal distributions. Finally, a clustering procedure was applied using the K-means technique to in order to detect different physiotherapist groups (clusters) using the scores of the HS-EBP five factors. Euclidean distance and a different number of clusters ranging from 2 to 6 were tested. All analyses were carried out using SPSS Statistics 25.0 (Chicago, IL, USA).

#### Ethical issues/statement

The study was approved by the Research Ethics Committee of the University of the Balearic Islands (registration n° 3566) and conducted according to the ethical guidelines of the Declaration of Helsinki; and data privacy was respected. Consent for participation was assumed when participants submitted their completed questionnaire online.

## Results

Out of the 901 participants who voluntarily entered the link enabled to answer the survey, 419 people completed it (46.5% response rate). Regarding sex, 58% were females (n = 243) and 42% males (n = 176), and non-statistically significant differences were obtained (p = .864) between female/male proportions of the sample and the reference population. Regarding age groups, 65.6% of physiotherapists in the sample were under 35 (n = 275), 28.2% were between 35 and 44 (n = 118), 4.5% between 45 and 54 (n = 19), 1.2% between 55 and 64 (n = 5), and 0.5% between 65 and 69 years (n = 2). The two-proportion comparison test between the sample and the target population was statistically non-significant for all age groups with p-values of .70, .89, .94, .98, and .97, respectively. The post hoc power of the test of proportions comparison was .91. Regarding academic degrees, 56.8% of physiotherapists had a diploma/bachelor’s/graduate degree, 38.2% had a master’s level, and the remaining 5% had a Ph.D. Concerning professional practice setting, 33.5% worked in Specialized Care Centers, 33.5% in their own practice, 14.7% in Socio-health Centers, 10.6% in Primary/Community Care Centers, 6% in a University, and 1.6% in the Education System. Mean weekly working hours was 37.44, with a standard deviation of 12.73 hours: males worked 40.14 hours weekly (SD = 12.73) and females 35.49 hours (SD = 12.39), with a statistically significant difference (t_417_ = 3.75, p < .001, Cohen’s d = 0.37). Regarding the diversity of tasks carried out, 69.74% of physiotherapists’ time was devoted to healthcare, 11.2% to management, 8.29% to teaching activities, and 7.99% to research; but differences by gender were non-significant. Finally, 50.8% of participants had received some EPB training whereas 49.2% had not, with non-significant differences between males and females.

The reliability indicators calculated from the latent structure of the HS-EBP questionnaire are provided in Table 1: Cronbach’s alpha (α), ranging from .80 to .96; Omega coefficient (ω), ranging from .80 to .96; Hierarchical Omega (ω_H_), ranging from .77 to .96; and the percentage explained variance (% EVA), which oscillated from 30.01% to 63.14%.

**Table 1:** Reliability indicators of HS-EBP questionnaire dimensions and % explained variance for each factor.

	Beliefs and attitudes F1	Results from literature F2	Professional practice F3	Assessment of Results F4	EBP Barriers/Facilitators F5
Cronbach’s Alpha (α)	.91	.96	.80	.94	.91
Omega (ω)	.92	.96	.80	.94	.91
Hierarchical Omega (ω_H_)	.92	.96	.77	.94	.90
% EVA	48.48	63.14	30.01	56.15	46.14

### Normative data

The results obtained in the questionnaire factors in the sample of physical therapists are shown in Table 2.

**Table 2:** Descriptive statistics of factor scores of the HS-EBP questionnaire from the Spanish physiotherapist sample.

	Beliefs and attitudes (F1)	Results from literature (F2)	Professional practice (F3)	Assessment of results (F4)	EBP Barriers/Facilitators (F5)
Mean (SD)^*^ **95% CI**	97.77 (13.88) [96.44–99.10]	92.28 (25.78) [89.80–94.75]	78.45 (11.28) [77.36–79.53]	86.05 (19.74) [84.15–87.95]	61.58 (23.82) [59.29–63.87]
**Mean (1–10) Median (P50)**	8.15 99.00	6.59 94.00	7.85 80.00	7.17 89.00	5.13 62.00
**P25-P75** ^**^	90.50–108.00	78.00–111.00	72.00–86.00	75.00–100.00	42.00–79.00
**Range**	25.00–120.00	20.00–140.00	11.00–100.00	16.00–120.00	12.00–120.00
**Skewness CI95%**	-0.807 (0.119) [-1.045; -0.569]	-0.391 (0.119) [-0.629; -0.153]	-0.753 (0.119) [-0.991; -0.515]	-0.655 (0.119) [-0.893; -0.417]	**0.086 (0.119) [-0.152; 0.324]**
**Kurtosis 95% CI**	0.959 (0.238) [0.483; 1.435]	**-0.303 (0.238) [-0.779; 0.173]**	1.213 (0.238) [0.737; 1.689]	**0.187 (0.238) [-0.289; 0.663]**	-0.775 (0.238) [-1.251; -0.299]
**K-S Test** ^***^	p<0.001	p<0.001	p<0.001	p<0.001	p = 0.001

^*^ SD = Standard deviation ^**^ Percentile 25-Percentile 75 ^***^ K-S test = Kolmogorov-Smirnov test with Lilliefors correction test

Non-significant 95% CI in bold

Normative data of the Spanish physiotherapist population for the HS-EBP five factor scores are shown in Table 3. The table provides the percentiles expressed by the total score and the average score on the item response scale (from 1 to 10) for each dimension, which facilitates the interpretation of the EBP level of any practicing Spanish physiotherapist assessed with the HS-EBP questionnaire by comparison with their reference population.

**Table 3:** Normative data of Spanish physiotherapist sample for the five factors of the HS-EBP questionnaire (total score and mean score of item response scale).

	Beliefs and attitudes F1		Results from literature F2		Professional practice F3		Assessment of Results F4		EBP Barriers/Facilitators F5	
	Total	Mean	Total	Mean	Total	Mean	Total	Mean	Total	Mean
Percentiles	12–120	1–10	14–140	1–10	10–100	1–10	12–120	1–10	12–120	1–10
**1**	56.40	4.70	29.00	2.07	41.80	4.18	29.80	2.48	16.20	1.35
**5**	72.00	6.00	45.00	3.21	58.00	5.80	45.00	3.75	26.00	2.17
**10**	79.00	6.58	53.00	3.79	64.00	6.40	58.00	4.83	29.00	2.42
**15**	83.00	6.92	62.00	4.43	68.00	6.80	67.00	5.58	34.00	2.83
**20**	87.00	7.25	71.00	5.07	70.00	7.00	70.00	5.83	37.00	3.08
**25**	90.00	7.50	78.00	5.57	72.00	7.20	75.00	6.25	42.00	3.50
**30**	93.00	7.75	82.00	5.86	73.00	7.30	77.00	6.42	46.00	3.83
**35**	95.00	7.92	85.00	6.07	75.00	7.50	81.00	6.75	51.00	4.25
**40**	96.00	8.00	88.00	6.29	77.00	7.70	84.00	7.00	55.00	4.58
**45**	98.00	8.17	91.00	6.50	78.00	7.80	86.00	7.17	58.00	4.83
**50**	99.00	8.25	94.00	6.71	80.00	8.00	89.00	7.42	62.00	5.17
**55**	101.00	8.42	97.00	6.93	81.00	8.10	91.00	7.58	65.00	5.42
**60**	103.00	8.58	101.00	7.21	82.00	8.20	94.00	7.83	69.00	5.75
**65**	104.00	8.67	104.00	7.43	83.00	8.30	96.00	8.00	72.00	6.00
**70**	106.00	8.83	106.00	7.57	85.00	8.50	98.00	8.17	76.00	6.33
**75**	108.00	9.00	111.00	7.93	86.00	8.60	100.00	8.33	79.00	6.58
**80**	109.00	9.08	115.00	8.21	88.00	8.80	102.00	8.50	83.00	6.92
**85**	111.00	9.25	119.00	8.50	90.00	9.00	106.00	8.83	88.00	7.33
**90**	114.00	9.50	125.00	8.93	92.00	9.20	110.00	9.17	93.00	7.75
**95**	119.00	9.92	133.00	9.50	95.00	9.50	115.00	9.58	101.00	8.42
**99**	120.00	10.00	140.00	10.00	100.00	10.00	120.00	10.00	112.80	9.40

#### Differential analyses

The results from applying differential analyses on the HS-EBP factor scores by sociodemographic and EBP-related variables are shown in Table 4.

**Table 4:** Differential analyses on the HS-EBP dimension scores from a normative sample of Spanish physiotherapists.

Variables	Beliefs and attitudes F1	Results from literature F2	Professional practice F3	Assessment of Results F4	EBP Facilitators F5
Sex					
a. Male (n = 176)	97.78	92.41	77.84	85.68	63.13
b. Female (n = 243)	97.76	92.19	78.89	86.32	60.46
Age	-.05	.02	-.01	.05	.06
Academic degree					
a. Bachelor’s (n = 238)	98.80	88.39abc**	77.56	83.25	56.60
b. Master’s (n = 160)	98.56	94.76abc**	79.28	88.77	63.29
c. Doctorate (n = 21)	102.67	117.48abc**	82.14	97.10	82.23
Years of practice	-.05	.01	-.01	.04	.07
EBP training					
a. Yes (n = 213)	100.44ab**	99.62ab**	80.67ab**	89.20ab**	65.37ab**
b. No (n = 206)	95.01ab**	84.69ab**	76.15ab**	82.80ab**	57.66ab**
EBP training level					
a. Basic (n = 55)	98.02	85.76abc**	78.20ac**	81.00ac**	61.40
b. Intermediate (n = 73)	99.74	96.21abc**	79.11bc**	88.49	62.04
c. Advanced (n = 85)	102.61	111.53abc**	83.60abc**	95.09ac**	70.79
Practice setting					
a. Rural (n = 35)	96.31	92.54	79.11	89.46	56.71
b. Urban <50,000 (n = 130)	97.49	91.55	77.94	87.49	60.51
c. Urban >50,000 (n = 254)	98.11	92.61	78.61	84.84	62.80
Main workplace					
a. Specialized Care (n = 123)	98.06	93.74ae**	78.30	83.50ae**	58.88aef**
b. Primary Care (n = 39)	100.26	91.00be**	76.87	83.03be**	59.34be**
c. Socio-health Center (n = 54)	95.98	91.78ce**	77.93	86.22	55.06cef**
d. School system (n = 6)	86.33	94.83	77.17	71.67de**	42.67
e. University staff (n = 22)	102.82	119.36abcf**	85.05	100.00abedf**	81.82abce**
f. On their own (n = 123)	97.39	90.12ef**	78.87	87.81ef**	68.99acf**
Type of organization					
Public (n = 108)	97.34	94.06	76.82	83.69	61.15
Private (n = 251)	97.77	91.67	79.00	87.23	62.90
Mixed (n = 52)	97.98	92.17	79.35	84.31	56.81
Weekly working hours (n = 419)	-.01	.11*	.04	.06	.08
Daily activities time % (n = 419)					
Healthcare	-.07	-.25**	-.11*	-.17**	-.26**
Research	.18**	.40**	.24**	.27**	.38**
Teaching	.04	.18**	.08	.11*	.21**
Management	-.02	-.07	-.05	-.03	-.07
Work alone					
a. Yes (n = 129)	97.20	90.85	76.17ab**	84.82	60.64
b. No (n = 290)	98.02	92.91	79.46ab**	86.60	61.99
Educational Center					
a. Yes (n = 153)	97.37	95.71b*	79.12	85.81	62.97
b. No (n = 266)	97.99	90.31a*	78.06	86.19	60.77
Student supervisor					
a. Yes (n = 131)	97.24	94.92	78.25	85.64	61.63
b. No (n = 288)	98.00	91.07	78.53	86.24	61.56

NOTE: The values of the means of the variables showing the same superscript are statistically significant (p < .05).

* = p < .05

** = p < .01

Statistically significant relationships were obtained concerning Academic degree (*Φc* = .207, *p* < .001), EBP training and EBP training level (*Φc* = .281, *p* < .001; and *Φc* = .292, *p* < .001; respectively), and main workplace (*Φc* = .187, *p* = < .001), with regard to the five HS-EBP factors.

Concerning academic degree, physiotherapists with a doctorate or a master’s obtained higher EBP scores than those with a bachelor’s for F2, F4, and F5, although differences were only statistically significant for F2. Physiotherapists trained in EBP scored significantly higher in all EBP factors, and significant differences were obtained between the three levels of training in EBP, especially for F2, F3, and F4; that is, the factors that operationalize the central EBP process: search for evidence in the literature, implementation in professional practice, and assessment of results. No significant differences were obtained in the factor scores of Attitudes towards EBP (F1) or Perception of barriers/facilitators (F5) by EBP training level.

Regarding the main workplace, physiotherapists in the University group obtained the highest scores in all HS-EBP factors, although they were only statistically significant for F2, F4, and F5. It is important to note that F1 and F3 scores did not show significant differences between any of the six levels of the "main workplace" variable. Correlations were statistically non-significant for physiotherapists’ age, number of years of practice, and all HS-EBP factor scores. A significant negative correlation was obtained between Healthcare and F2 (-.25), F3 (-.11), F4 (-.17), and F5 (-.26) scores, in terms of the percentage of time spent on daily activities; while a significant positive correlation was found between Research and all EBP factors: F1 (.18), F2 (.40), F3 (.24), F4 (.27), and F5 (.38). Finally, working alone, working in an educational center, or being a student supervisor were statistically non-significant variables over HS-EBP factor scores.

### EBP profiles by clusters

Six different clusters were detected as the optimal grouping solution using the HS-EBP five dimension scores (Table 5): Group 1 (G1), with low scores in all factors; Group 2 (G2), with low scores in all factors but with medium scores in F3 and F4; Group 3 (G3), with medium scores in all factors, but with low scores in F5; Group 4 (G4), with medium-high scores in all factors, but with low scores in F5; Group 5 (G5), with medium-high scores in all factors; and Group 6 (G6) with high scores in all factors.

**Table 5:** Clusters detected using the HS-EBP dimension scores (total sum and 1–10 scale).

Clusters	Beliefs and attitudes (F1)		Results from literature (F2)		Professional practice (F3)		Assessment of Results (F4)		Barriers/Facilitators (F5)	
	12–120	1–10	14–140	1–10	10–100	1–10	12–120	1–10	12–120	1–10
**Cluster1** (n = 46)	87.87	7.32	55.07	3.93	62.76	6.28	49.65	4.14	40.89	3.41
**Cluster2** (n = 51)	86.49	7.21	57.53	4.11	74.25	7.43	83.98	7.00	44.47	3.71
**Cluster3** (n = 67)	98.87	8.24	89.33	6.38	73.94	7.39	72.15	6.01	48.18	4.02
**Cluster4** (n = 63)	100.35	8.36	105.35	7.53	82.56	8.26	96.49	8.04	41.37	3.45
**Cluster5** (n = 116)	96.41	8.03	96.01	6.86	79.01	7.90	89.61	7.47	77.14	6.43
**Cluster6** (n = 76)	110.30	9.19	124.20	8.87	90.46	9.05	107.63	8.97	90.39	7.53
**Total** (n = 419)	**97.77**	**8.15**	**92.28**	**6.59**	**78.45**	**7.85**	**86.05**	**7.17**	**61.58**	**5.13**

The first and last cluster, respectively, achieved the highest and lowest scores in all EBP factors. The first two clusters presented similar low scores for F1, F2, and F5; whereas Cluster 2 showed better results for F3 and F4. The fundamental difference between intermediate Clusters 3 and 4, and low Clusters 1 and 2, is that the former obtained much higher scores for F1 and F2 –that is, for EBP beliefs and attitudes and literature evidence search–and similar scores for the other factors, especially F5. Finally, what differentiates intermediate Clusters 3 and 4 from Clusters 5 and 6 is that the latter presented higher scores for F5 –that is, this cluster of physiotherapists perceives fewer barriers to EBP in their daily practice.

If we consider the percentage of work time spent on daily activities such as healthcare, clinical research, or teaching, together with the number of weekly working hours, the structure of the different clusters detected becomes more understandable (Table 6).

**Table 6:** One-way ANOVA with post-hoc Bonferroni test to compare the means of the total scores of each of the 6 categories (clusters) concerning the % of time spent on daily practice activities, and weekly working hours.

		Cluster 1 (Low)	Cluster 2	Cluster 3	Cluster 4	Cluster 5	Cluster 6 (High)
**% of working time spent on different daily activities**	**Healthcare**	79.22^ae^	74.22^b^	72.34^c^	75.76^d^	66.91^e^	58.03^abcd^
	**Research**	2.74^af^	3.12^bg^	6.87^c^	5.19^d^	9.12^efg^	16.01^abcde^
	**Teaching**	4.89^a^	5.18^b^	7.58^c^	6.35^d^	8.73	13.97^abcd^
**Weekly working hours**		40.11^a^	31.45^abc^	37.04	39.29^b^	37.22	39.01^c^

NOTE: Values of cluster means (named from “a” to “f”) showing the same superscript are statistically significant (p < .05).

**Fig 1:**
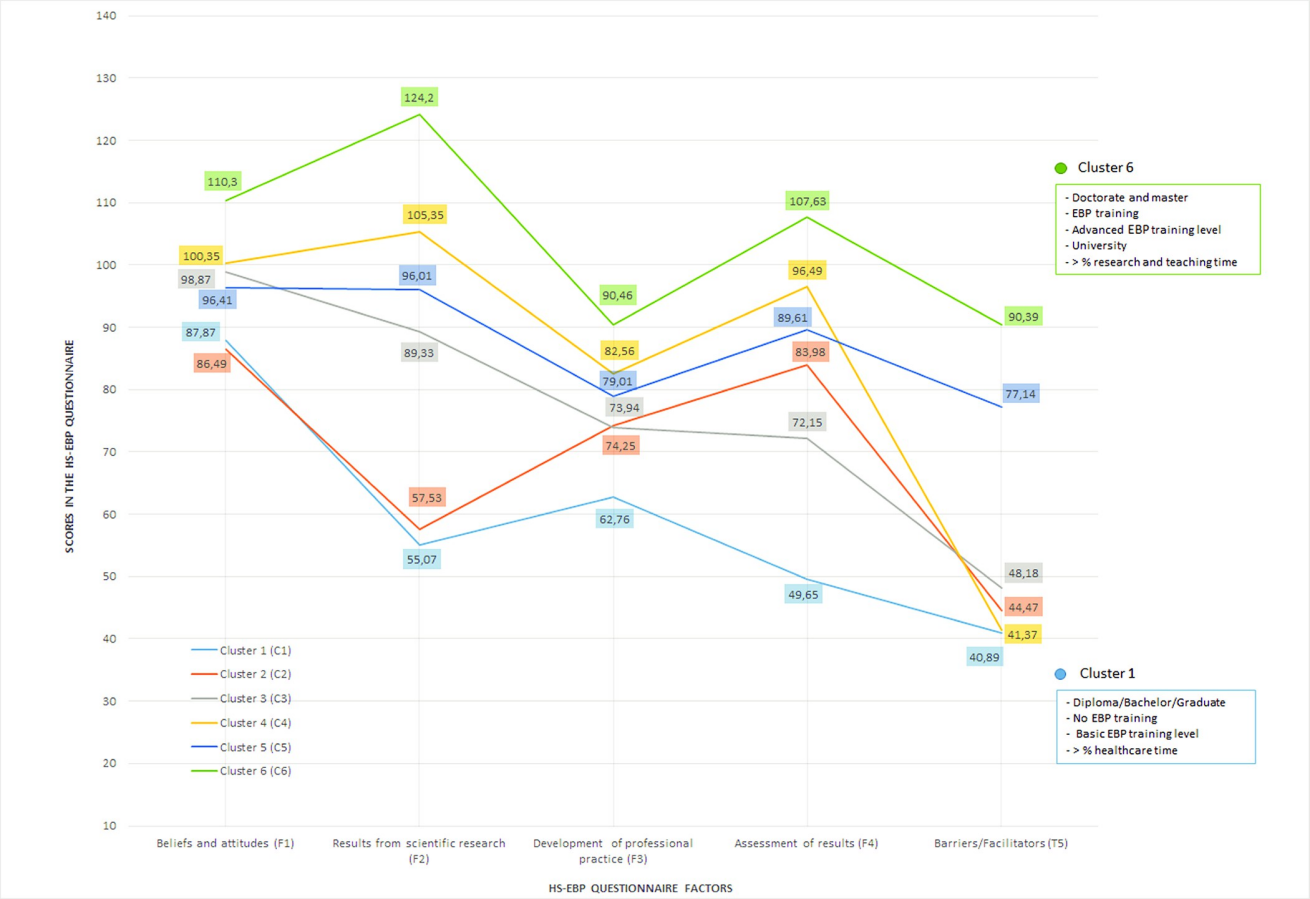
Profile of scores (means) in the five factors of the HS-EBP questionnaire by cluster. NOTES: 1. The figures included in the table reflect the mean score obtained by the subjects belonging to each of the clusters in each of the five factors of the HS-EBP measurement questionnaire, 2. Categories of sociodemographic, training and/or practice variables for which significant profile differences were found in the C6 and C1 clusters can be seen in the right margin of the table, 3. Range of possible scores in each of the factors of the HS-EBP questionnaire: from 12 to 120 in F1, F4, and F5; 14–140 in F2; and 10–100 in F3.

Cluster 1 showed the highest percentage of time spent on healthcare (79.22%) and the lowest for research (2.74%) and teaching (4.89%), while Cluster 6 revealed the lowest percentage of time spent on healthcare (58.03%) but the highest on research (16%) and teaching (13.97%)–that is, the more time spent on research and teaching, the higher the scores in EBP factors. However, scores decreased significantly as time spent on health care increases. Number of working hours per week obtained significant effects for cluster differentiation but with a smaller impact and a more diffuse pattern. There were no significant differences between cluster 1 (40.11 hours; more healthcare) and Cluster 6 (39.01 hours; more research and teaching); while the main differences corresponded to Cluster 2, with the lowest weekly dedication (31.45 hours). Fig 1 depicts each EBP cluster profile according to HS-EBP factors and includes the variables with a significant effect on the differences between Cluster 1 (low EBP scores) and Cluster 6 (high EBP scores).

## Discussion

The HS-EBP questionnaire previously obtained adequate reliability and evidence of validity (content, latent structure, and criteria). However, an exhaustive CTT psychometric validation process also requires normative data from the reference population to be obtained. Therefore, the main goal of this study was to obtain normative data from the Spanish Physiotherapist population using the HS-EBP.

Regarding EBP normative data from healthcare professionals, there are only a few recently published studies on EBP measurement tools that provide normative data analysis: specifically, the Finnish version of the Steven´s Evidence Based Practice Readiness Inventory [19] with 943 hospital nurses, and the Polish version of the EBP Competency Questionnaire [20] with 1636 nursing students. To our knowledge, the HS-EBP questionnaire is the only instrument on EBP developed in Spain in which normative data have been obtained, specifically until now from osteopathy professionals as a reference population [21]. This normative information enables each individual’s test score in all HS-EBP dimensions to be compared to the score distribution (percentile scores) of their professional reference population, serving as a reference point to compare and interpret individual scores obtained using the HS-EBP questionnaire. In addition, normative data make it possible to describe differential EBP patterns using the scores obtained through the factors of the test latent structure and also to detect EBP shortcomings, which enables a start-point to be established so as to improve EBP implementation.

A representative sample of the general practicing population of physiotherapists in Spain is needed in order to obtain adequate normative data. The data for describing Physiotherapy practice are only available from the survey published by the Spanish National Statistical Institute, with the latest survey corresponding to 2018 [17]. The present study did not apply a stratified random sampling strategy as the optimal way because it would only have been feasible if the random selection of registered Physiotherapists and the sending of the questionnaires had been carried out by each regional delegation of the Spanish Professional Physiotherapy Council initially selected. However, this was not possible given the limited human resources available and the existing infrastructure in these delegations. Faced with the impossibility of implementing an optimal sampling strategy, we decided to use a non-probabilistic procedure, so the participating delegations disseminated the link to the survey among their members and encouraged them to answer it. Despite this limitation, the sample was representative enough of the reference population by both sex and age group.

After verifying that the representativeness of the sample was reasonable despite not being probabilistic, reliability coefficients from the questionnaire’s latent structure were estimated. All reliability values were adequate, ranging from .90 to .96 except for Factor 3 (.77), which was very near the cutoff (greater than or equal to .80). According to these values, the percentages of explained variance by HS-EBP factor were satisfactory, close to or greater than 50%, except for Factor 3 (30.01%). These results for Factor 3 are similar to those obtained by the original validation study [12]. This psychometric behavior may be due to difficulties in measuring the implementation of evidence (EBP practice) and to a lower number of factor items (10) compared to the rest of the HS-EBP factors. Improvement in the psychometric behavior of Factor 3 is one of the fundamental lines of work to be developed on to create version 2.0 of the HS-EBP instrument. Either way, these results are adequate enough so as not to invalidate their use and interpretation.

Concerning HS-EBP normative data, the 50^th^ percentile values by factors (scale 1–10) were, in decreasing order, 8.25 (Factor 1), 8.00 (Factor 3), 7.42 (Factor 4), 6.71 (Factor 2), and 5.17 (Factor 5). These average normative values describe the Spanish physiotherapist population as showing strong positive attitudes and beliefs regarding EBP (F1) [22], a similar level of evidence implementation in daily practice (F3), and slightly lower but appropriate assessment of results (F4). Factor 2, searching for literature evidence, and Factor 5, perception of organizational factors as barriers or facilitators for EBP implementation, show lower values, particularly F5. These results are in line with the literature that concludes that health professionals perceive organizational factors as the main barriers to implementing EBP [23]–that is, aspects related to the resources and material support provided by the organization for accessing EBP (such as institutional access, infrastructures, access to training, and support resources); as well as resources and human support and, also, the organizational culture towards EBP promotion [18]. In this study, the main barriers perceived by Spanish physiotherapists are that the application of EBP is not encouraged/rewarded (Item 11 = 3.63), the patients do not demand their treatments be evidence-based (Item 7 = 3.65), the time distribution of the workday does not facilitate the search for and application of scientific evidence (Item 10 = 4.04), and there is a lack of enough recommendations or demands in the work environment for the use of EBP (Item 9 = 4.59). However, the elements perceived as facilitators for EBP by Physical therapists are that most of their colleagues in Physiotherapy (Item 5 = 6.32) and also from different professions (Item 6 = 6.21) have a favorable attitude towards using results from research in their practice, access to resources related to scientific evidence (Item 1 = 6.21), and that it is a priority to be kept up-to-date with the results from research (Item 3 = 6.01).

According to the normative data for Factor 5 (Table 3), only 10% of physical therapists perceive a good context for implementing EBP (90^th^ Percentile = 7.75) with the 50^th^ percentile scoring only 5.17. These results indicate the need to reduce barriers to EBP, especially in terms of promoting/reinforcing its application by supervision and management agents, promoting its use in the work environment by establishing clear guidelines or recommendations, improving time distribution and management, and providing information and advice for encouraging patients to request evidence-based treatments.

Therefore, based on the results obtained in this study using the HS-EBP questionnaire as a measurement instrument for the main EBP theoretical domains, the implementation of EBP is already and should continue to be one of the cornerstones of evidence-based physical therapy research, with the aim of eliminating the barriers detected and improving the specific facilitators of EBP in clinical settings by implementing training programs tailored to the specific shortcomings found.

The differential analyses concerning normative scores using EBP-related variables showed statistically significant differences only for academic degree, EBP training, main workplace, and percentage of time spent on different daily activities by physiotherapists. Regarding the three levels of academic degree, there are significant differences in searching for evidence in the literature: holders of a Ph.D. presented a higher score than those with a Master’s Degree, and these, in turn, were higher than BSc scores. However, differences were not significant for the rest of the HS-EBP factors. This result coincides with the conclusions of various studies on educational level and EBP, specifically concerning the search for evidence in the literature [5, 24, 25].

Meanwhile, similar to other studies [25], levels of EBP training generate significant differences in most behavioral factors of EBP (Factors 2, 3, and 4)–searching for existing evidence, implementing it, and assessing the results–factors that represent the necessary steps for the appropriate application of EBP that should be carried out routinely in each clinical encounter [26]. Scores on these factors are higher as the level of EBP training increases (Basic, Intermediate, and Advanced). However, EBP training level has no significant effect on the scores for Factor 1 (beliefs and attitudes towards EBP) or Factor 5 (the perception of barriers and facilitators towards EBP). These results are in line with different intervention studies, demonstrating that workshops or other types of professional development, training, and education focused on EBP can have a positive effect on increasing skills, knowledge, attitudes, and behaviors, with regard to EBP.

Working in an academic environment or spending more daily time on research-related tasks represents higher scores for behavioral EBP factors and lower scores of perception of barriers to EBP implementation [25, 27]. More time spent on direct healthcare tasks with patients is associated with worse attitudes and beliefs towards EBP and the perception of more barriers to implementing it, consequently, with less application of the EBP process in Spanish physiotherapists’ daily clinical practice.

Although some studies have found significant associations between EBP and age [4, 5, 7, 28–30], years of professional experience [4, 25, 31–33], working in a multidisciplinary team [29], number of people in the workplace [4, 29], years of practice [7, 25, 32, 33], and work settings [25, 31], these variables did not show significant effects in the population of Spanish physiotherapists.

According to these findings, obtaining higher scores for the HS-EBP factors is related to working in an academic environment (University) which, in turn, requires having a higher academic degree, more amount of time spent on teaching and research activities, and better EBP training. Meanwhile, the burden associated with patient care in daily practice might be acting as a barrier to EBP process implementation. Clinical physiotherapists generally obtained lower scores for all HS-EBP factors except for beliefs and attitudes towards EBP; as they probably understand the importance of implementing existing evidence and have positive beliefs and attitudes, but at the same time perceive more EBP barriers, and find it more difficult to search the literature, implement the existing evidence, and assess the results. Similar results have been obtained in some studies in clinical Physiotherapists conducted in different countries worldwide [4, 6, 7, 25, 29, 30, 34–37].

Finally, another objective of the study was to explore the existence of clusters that represent different EBP patterns for the Spanish physiotherapist population using the HS-EBP questionnaire factors’ normative scores. The optimal solution presented a structure of 6 clusters after estimating different configurations.

Only two clusters showed a uniform pattern of high scores (G6) and low scores (G1) across the five EBP factors analyzed, as depicted in Fig 1. This structure of clusters enables the results obtained to be better visualized than previously using differential analysis. The clusters obtaining low EBP scores (G1 and G2) include physiotherapists working in clinical healthcare, whereas the clusters obtaining high EBP scores (G5 and G6) include those working in teaching and/or research. However, weekly working hours do not show statistically significant correlations between the factor scores of the clusters. Therefore, obtaining better EBP scores does not depend so much on the amount of time worked as on the type of work performed by physiotherapists.

Information on the characteristics of G6 physiotherapists can be useful for implementing EBP strategies based on both mentorship support and knowledge spreading leadership [27, 38, 39]. The G1 cluster represents physiotherapists with a high risk of not including EBP principles in their current clinical practice. Individualized diagnoses of behavior and/or competence in EBP would facilitate decision-making regarding the development and implementation of intervention programs. The real challenge is to make healthcare compatible with the application of EBP, because the main goal is to improve interventions with patients based on evidence.

The rest of the clusters follow a non-uniform pattern of scores throughout the EBP factors. It is only worth noting that when compared to clusters with lower or higher scores, the scores in the significant variables follow the same trend observed between the extremes. An advantage of each cluster presenting similar scores across the HS-EBP factors is that the application of EBP intervention programs would produce similar improvements simultaneously in all of them.

To our knowledge, there are currently no published studies that analyze potential variables that could influence the adoption of EBP from normative data using a cluster approach.

By way of limitations of the study, these results must be interpreted carefully for three reasons: 1) despite the representativeness of the sample for sex and age groups in the reference population having been verified, the normative (not probabilistic) sample might be made up of physiotherapists with a propensity for EBP (self-selection bias); 2) the self-reported measures could have been affected by social desirability; and 3) these normative data are only a reflection of the current state of EBP Physiotherapy in Spain. If the self-selection bias is operating, an overestimation effect of the results obtained for the different EBP factors may have been produced, especially for items related to Factor 1 (Beliefs and attitudes towards EBP).

## Conclusions

This study provides normative data regarding the Spanish Physiotherapist population using the HS-EBP questionnaire. The reference values obtained reveal that Spanish physiotherapists show strong positive attitudes and beliefs about EBP, and that organizational factors are considered the main barrier to EBP implementation, especially for professionals focused on clinical healthcare activity.

Differential analysis using some sociodemographic, training, and practice variables, together with the development of an analysis of EBP profiles through clustering techniques, reveals that the variables with the greatest impact on the scores of the factors of the HS-EBP questionnaire are: academic degree, being involved in teaching and research activities, and working in an academic environment. No significant associations were found between EBP factor scores and sociodemographic variables (sex and age), practice-specific features such as management activity, years of professional practice, or weekly working hours.

Having EBP normative data from the Spanish physiotherapist population makes it possible to interpret the HS-EBP scores of any individual professional. The table of normative data provided by this study can be considered the first contribution in Spain to the EBP measurement of Physiotherapy. This information can be useful for developing intervention programs to encourage EBP implementation in physiotherapists’ daily clinical practice. It is important to note that assessing professional EBP using a validated tool like the HS-EBP questionnaire helps fight against the use of pseudo-therapies, especially in Physiotherapy.

## Supporting information

S1 Checklist Checklist for Reporting Results of Internet E-Surveys (CHERRIES). (DOCX)

S2 Checklist STROBE statement—checklist of items that should be included in reports of *cross-sectional studies*. (DOCX)

S1 File (SAV)
